# The Public's Preferences for Psychological Interventions During the COVID-19 Pandemic: A Discrete Choice Experiment

**DOI:** 10.3389/fpsyt.2022.805512

**Published:** 2022-04-27

**Authors:** Hui-qin Li, Shu-xiang Liu, Hui Xue, Hua Yuan, Xiu-ying Zhang

**Affiliations:** ^1^Department of Fundamental Nursing, School of Nursing, Jilin University, Changchun, China; ^2^Department of Histology and Embryology, College of Basic Medical Sciences, Jilin University, Changchun, China

**Keywords:** public health, psychological health, health care, health policy, preferences

## Abstract

**Aims:**

To explore the public's preference for psychological interventions through a discrete choice experiment and to provide references for formulating psychological intervention policies and establishing psychological intervention procedures in response to public health emergencies.

**Methods:**

This study is a discrete choice experiment. Attributes and levels were identified through literature reviews, in-depth interviews, focus group discussions, and expert consultations. Experimental design principles were applied to generate choice sets containing different attribute levels and develop a survey instrument. Convenience sampling was conducted nationwide, and 1,045 participants were investigated. A mixed logit model was used to evaluate the public's preferences.

**Results:**

All attributes in our study were found to have a significant influence on the public's preferences for psychological interventions during the COVID-19 pandemic. The public's preferences for providers and duration were influenced by the public's levels of education and classifications. Furthermore, the most ideal scenario was found to be a one-on-one psychological intervention provided by family and friends through social network platforms, for which the frequency is twice per week, and the duration of each intervention is 0.5–1 h.

**Conclusions:**

The public's preferences for psychological interventions during the COVID-19 pandemic are affected by the method, form, frequency, provider, and duration of interventions. Our findings provide references for the formulation of psychological intervention policies and the establishment of psychological intervention procedures in response to public health emergencies.

## Introduction

COVID-19 has greatly endangered the health and life safety of the public and attracted attention from all countries and regions. According to a report from the World Health Organization (WHO) on 1 February 2022, the number of people infected with COVID-19 has exceeded 376 million, and the number of deaths totals 5.6 million (https://www.who.int/emergencies/diseases/novel-coronavirus-2019/situation-reports/). At present, home quarantine is the main means through which to prevent COVID-19 infection and the spread of the pandemic. However, the loss of face-to-face communication and other regular social interventions caused by quarantine have made the public experience stressful situations (1), and such short-term stressful situations may develop into adaptation disorders and posttraumatic stress disorder (PTSD) (2). In addition, during the COVID-19 pandemic, the threat of disease and the economic burden caused by the suspension of work have had a negative impact on the public's mental and psychological states, which have manifested as anxiety, depression, and stress (3–5). Many studies have assessed the psychological impact of COVID-19 and found high levels of psychological distress (6–12). Furthermore, the overflow of information about the COVID-19 pandemic has also triggered public panic, which may lead to extreme behaviors such as suicide (13). Therefore, it is necessary to provide effective psychological interventions for the public to prevent and/or alleviate mental and psychological problems.

Faced with the COVID-19 pandemic, various regions in China have implemented corresponding psychological interventions. However, some medical staff are unwilling to accept the current psychological interventions provided by some teams or individuals (14). Furthermore, some researchers have claimed that the mental health needs of COVID-19 patients, suspected patients with COVID-19, quarantined family members, and medical personnel have been poorly handled (5), which may be due to a lack of understanding about the public's mental health needs and preferences for psychological interventions. Understanding the public's preferences for psychological interventions is conducive to the formulation of more acceptable and targeted psychological intervention strategies to improve the effectiveness of such interventions.

A discrete choice experiment (DCE) is the most common and main preference measurement method (15); it can not only calculate the regression coefficient and willingness to pay (WTP) to reflect people's preferences but also simulate the influence of changes in influencing factors on these preferences (16, 17). In the field of health psychology, DCEs are often used to design patient-centered psychological care measures. Goodall et al. conducted a DCE to determine the preferred characteristics of psychosocial support services for adolescents and young people with cancer or blood diseases and their caregivers (18). Herman et al. used DCE to explore patients' preferences for mental health services provided to low-income Hispanics engaged in primary care (19). Lokkerbol et al. used a DCE to assess the preferences of patients with depression and anxiety for psychotherapy (20, 21). However, no research has explored the public's preference for psychological interventions during COVID-19 pandemic public health emergencies to provide a reference for the formulation of such intervention programs. Therefore, the purpose of this study is to explore the public's preference for psychological interventions during the COVID-19 pandemic through a DCE to provide a reference for the formulation of psychological intervention policies and the establishment of psychological intervention procedures in response to public health emergencies and to provide references for randomized controlled experiments to explore the differences in the effects of psychological interventions during public health emergencies.

## Methods

### Design

**Figure 1:**
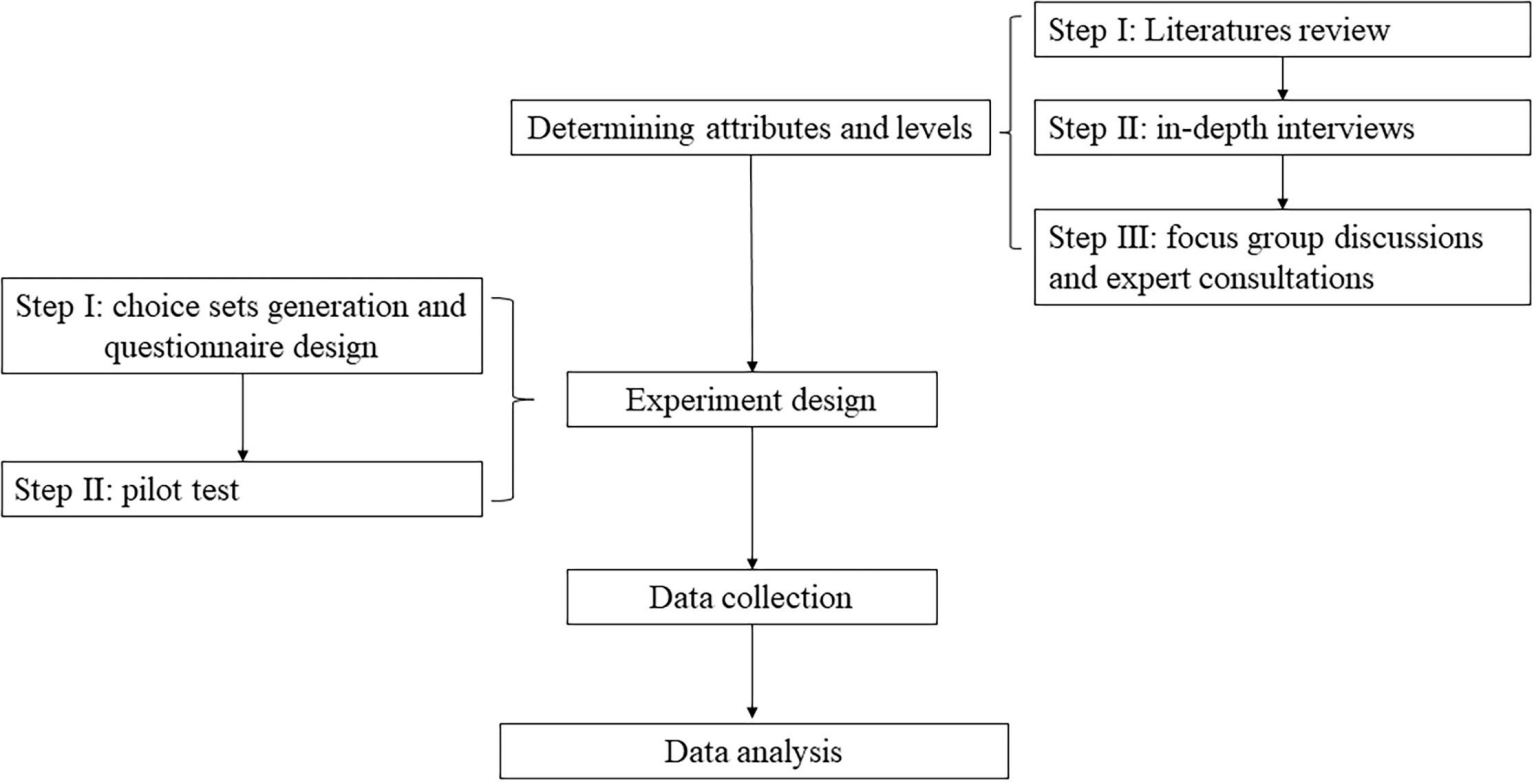
The process of DCE.

This study used a DCE approach to understand the public's preferences for psychological interventions in COVID-19 pandemic public health emergencies. The main processes of this DCE include determining attributes and levels, experimental design, data collection, and data analysis, the details of which are shown in Figure 1.

### Determining Attributes and Levels

#### Step I: Literature Review

We determined attributes and levels based on published recommendations (22, 23). First, the literature was searched through electronic databases, such as CNKI, Wanfang Database, PubMed, Web of Science, and Embase, and full-text articles, available before 31 July 2020, were reviewed. The search strategy was “COVID-19” OR “public health emergencies” AND “mentality” OR “psychology” OR “psychic.” Then, we extracted the psychological status and its influencing factors of the public under COVID-19 and public health emergencies, the public's needs and expectations for psychological interventions, and the factors affecting the public's acceptance of psychological interventions. We identified 6 potential attributes based on the literature review, which we discuss later in our qualitative study.

#### Step II: In-Depth Interviews

Based on the literature review, an interview outline was developed, and one-to-one in-depth interviews were conducted by telephone due to the impact of COVID-19. The interview outlines were as follows: (1) the current psychological state of the interviewees, (2) the currently available psychological intervention strategies, (3) the accessibility of psychological intervention services, (4) the availability of emotional or economic resources, (5) the need for psychological intervention during the COVID-19 pandemic, and (6) interviewees' attitudes and suggestions concerning psychological intervention during COVID-19 and public health emergencies. Using purposive sampling, interviewees were chosen according to their location, age, and education level. Interviews were carried out until their content reached saturation. All interview data of the 12 interviewees were recorded and transcribed verbatim and analyzed with NVivo 12.0. Eventually, the list of potential attributes was expanded to nine, namely, place, mode, frequency, form, provider, continuity, content, total length of time of instruction, and duration of each instruction.

#### Step III: Focus Group Discussions and Expert Consultations

Focus group discussions were conducted by video conference after the in-depth interviews. Fifteen participants were included based on different regions, educational backgrounds, ages, and exposure to COVID-19, and they were randomly divided into 3 groups, with 5 participants in each group. During each discussion, participants were provided with 9 attributes obtained from the literature review and in-depth interviews and asked to add new attributes and discuss the definition of these attributes and levels until they reached a consensus. Next, one-to-one expert consultations in Deyang city, Sichuan province, were conducted face to face. Experts included a health department staff member, a psychologist, and a doctor, all with more than 10 years of work experience, who were asked to add new attributes and revise inappropriate attributes, which ensured that the potential attributes and corresponding levels were appropriate under the current policy and medical background. Then, 15 respondents who participated in the focus group discussion were contacted *via* WeChat (a Chinese online social network similar to Facebook) and were asked to vote for each attribute with “most,” “somewhat,” and “least.” According to the number of “most” votes, the attributes were sorted. In the field of health care, the number of attributes in most DCEs is 4–9, and the median number of attributes is 5 (24). Therefore, the top five attributes were included in this study, and their levels were developed, which are method, form, frequency, provider, and duration (refer to Table 1 for details).

**Table 1:** Attributes and levels.

**Attributes**	**Level**	**Description**
Method	Face to face	A visit to a provider of psychological intervention where you would have a psychiatric evaluation and discussion about your mental and psychological issues.
	Phone	At a scheduled time, a provider of psychological intervention telephones you and you have a discussion about your mental and psychological issues and provides guidance and interventions on these issues.
	Social network platform	At a scheduled time, a provider of psychological interventions uploads mental health articles and videos on the Internet platform, or provides psychological guidance and interventions through social platforms such as WeChat and QQ.
Form	One to one	During the psychological intervention, a provider speaks to you individually about your feeling or opinions, or ask you questions about your mental and psychology, and provides guidance and interventions to you.
	One to many	During the psychological intervention, a provider speaks about public mental and psychology feeling, and provides guidance and interventions to other people besides you at the same time.
Frequency	Twice per week	Psychological interventions were provided twice a week.
	once per week	Psychological interventions were provided once a week.
	Once every 2 weeks	Psychological interventions were provided once every 2 weeks.
	No fixed time	Psychological guidance and interventions can be provided when you need them.
Provider	Psychologist	People who majors in psychology studies the human mind and tries to explain why people behave in the way that they do.
	Medical staff	Doctors or nurses who have undergone additional training in Psychological assessment, counseling and interventions
	Family and friends	Your family and friends who have received trainings about psychological knowledge.
	Volunteer	People who volunteer to participate in the prevention and control of COVID-19 and have undergone additional training in psychological assessment, counseling and intervention.
Duration, hours	<0.5	The duration of each psychological intervention was less than half an hour.
	0.5–1	The duration of each psychological intervention is between half an hour and an hour.
	≥1	The duration of each psychological intervention was more than 1 h.

### Experimental Design

#### Step I: Choice Set Generation and Questionnaire Design

In our study, three attributes have four levels, one attribute has three levels, and one attribute has two levels. According to the full factorial design, 384 (4^3^ × 3 × 2 = 384) possible scenarios were generated, which in turn generated 1,47,072 (384 × 383) possible choice sets. The existence of too many choice sets results in respondents' high cognitive burden and consumes considerable labor, material resources, and time (25). Therefore, the fractional factorial design was needed to reduce choice sets down to a manageable level. In a DCE, the commonly used fractional factorial design mainly includes orthogonal design and efficiency design. The Ngene 1.2 USER MANUAL & REFERENCE GUIDE (http://www.choice-metrics.com) shows that an efficient design always outperforms an orthogonal design in the case of any information about the prior parameters (even if this information involves only the sign of the prior parameter), where the sign of the parameter can be known by reasoning alone, and a slight positive or negative value can improve the design. In our study, a D-efficient design was carried out in the Ngene software to generate the choice sets, in which a slight prior parameter value was added for each attribute and was adjusted several times to minimize the D-error value. Finally, 16 different choice sets composed of attributes and levels were generated. In the field of health care, the choice sets of a DCE usually total 8 (26). Thus, the 16 choice sets were randomly divided into two versions to further reduce the burden on respondents. To test the corresponding consistency, the second choice set in each version was repeatedly included as the ninth choice.

At the beginning of the questionnaire, the purpose of this study, the contents of the questionnaire, and the requirements for filling in the questionnaire were introduced. The first part of the questionnaire is a general data questionnaire, which includes sociodemographic characteristics such as gender, age, income, level of education, and classification of population. The second part is the DCE questionnaire, which contains nine choice tasks, each of which contains two alternatives and one exit item. In this section, the attributes and levels are described, and an example of a choice set is provided (e.g., refer to Box 1). Then, respondents were asked to select their most preferred option in each choice set.

Box 1 **Description and an example of choice set.**

You will be asked to answer nine questions about hypothetical psychological intervention Programs. Each questions contains two alternatives and an exit option for you to choose and each question can only choose one option. The features of the psychological intervention programs will differ in the following five aspects:

Method: How you mental health information and psychological guidance.

Form: In what from does the provider provide you will psychological intervention.

Frequency: How often appointments of psychological intervention would be.

Provider: Who provides you for psychological interventions.

Duration: Duration of each psychological intervention.

An example of choice set

**Table:** 

**Attributes**	**Programme A**	**Programme B**
Method	Social network platform	Face to face
Form	One to one	One to many
Frequency	Random	Once every two weeks
Provider	Psychologist	Medical staff
Duration (h)	0.5–1	≥1

Which programme do you prefer:

Programme A Programme B Unwilling to receive psychological intervention.

#### Step II: Pilot Test

A pilot test was conducted among 50 respondents (25 respondents in each of the two versions). Most respondents who participated in the pilot test considered the question length of the questionnaire to be “acceptable,” “easy to understand,” and “appropriate,” and “the text is clear and easy to understand”; we revised the wording to improve the clarity of the questionnaire based on feedback from some of the 50 participants.

### Participants

Nationwide convenience sampling was used to recruit eligible participants. Individuals with reading and comprehension abilities were considered potential participants of our study. At the same time, people with cognitive impairment, people who could not complete the survey due to certain reasons, people affected by psychiatric illnesses, and people who were unwilling to participate in this study were excluded. According to Johnson (27) and Orme (28), the calculation formula of the minimum sample size N is as follows:


n > 500c/(t×a)

In this equation, *t* is the number of choice sets faced by an individual (excluding the choice set repeatedly included), *a* is the number of alternatives in each choice set (excluding exit items), and *c* is the number of analysis cells (when considering the main effect, *c* is equal to the maximum level number of any attribute). The minimum sample size needed in each version of the questionnaire is 125 (*t* = 8, *a* = 2, *c* = 4). We plan to mark the two versions of the questionnaire with 1 and 2. Considering that 30% of the recovered questionnaires may be invalid, the total sample size is 358 participants to ensure that sufficient data are included in the analysis and to obtain wide representativeness.

### Data Collection

Data were collected by conducting a questionnaire survey, which was performed by trained researchers. Participants were provided with hard-copy questionnaires as a priority, and for those participants who were not convenient to obtain a hard copy, electronic questionnaires were provided *via* WeChat or email. All questionnaires were completed by the participants themselves. In the questionnaires distributed, the versions were random, and the number of each version was the same. The data collection period was from 20 August 2020 to 25 November 2020.

### Data Analysis

Data were double entered into Epidata 3.1 and transferred to Stata 15.0 for processing and analysis. Descriptive statistics were reported for participants' sociodemographic characteristics. A mixed logit model was used to evaluate the preferences of participants for the different levels of the psychological intervention attributes. The use of a mixed logit model makes it possible to explore the preference heterogeneity of respondents (29–31) and allows for multiple observations from each respondent who was presented with nine choice sets. All models included main effects without interaction terms. All variables were coded as dummy variables to better reflect their influence on respondents' preferences.

The main output of the mixed logit model is an estimation of the proportion of respondents who prefer each attribute level compared with the reference level for each attribute. For instance, for the attribute “method,” the proportion of respondents preferring to intervene through social network platforms compared with face-to-face intervention can be estimated. A negative (positive) parameter sign indicates that the attribute level is not preferred (preferred) to the reference level of the attribute.

Adverse mental health status during the COVID-19 pandemic, such as stress, anxiety, and depression, has been affected by educational attainment (32). People with different educational attainment levels may have different needs for psychological interventions. Furthermore, the psychological pressure placed on people with different exposures to COVID-19 may also be different; thus, their needs for psychological intervention may also be different. Therefore, subgroup analysis was conducted based on levels of education and classifications of the population.

The sum of the model coefficients for each combination of attribute levels is the preference score (*V*_*j*_), which is also known as the indirect utility score. *P*_*j*_ represents the probability that each combination of attribute levels is the most preferred scenario, the calculation formula of which is as follows:


$$P_{j}=\frac{exp(V_{j)}}{\sum_{k=1}^{J}exp(V_{k)}}$$

where *j* =1, …, *J*. In this article, only the top five scenarios with the highest rankings are considered.

### Ethical Considerations

This study was approved by the University Ethics Committee and all other relevant organizations. Before the investigation began, the purposes of the study were explained to participants, and their informed consent was obtained. Furthermore, all information was anonymized, all data were used for research purposes only, and participants had the right to withdraw from the study at any time.

### Validity and Rigor

Two people cross-checked the questionnaire for quality control to ensure the validity of the data. Invalid questionnaires were defined as follows and were excluded: questionnaires that (1) had not been completed, (2) failed the consistency test, (3) had the same options checked in the entire questionnaire, and (4) had regularly checked items in the questionnaire.

## Results

### Characteristics of Respondents

A total of 1,200 people accessed the survey, 92 of whom did not complete the questionnaire and 63 of whom did not pass the consistency test. Finally, 1,045 people were included in the analysis, and the response rate was 87.08%. Among the 1,045 participants, 507 were men (48.52%), 538 were women (51.48%), and the majority of the respondents were between 20 and 59 years old (74.06%, which is equal to the sum of the proportions of those aged 20–39 and 40–59 years, which is 36.94 and 37.12%, respectively). The urban population accounted for 64.50%, and 53.11% of the respondents had a secondary school education (including junior high school and high school). Most people belonged to the third and fourth classifications, accounting for 34.74 and 34.35%, respectively. More details are presented in Table 2.

**Table 2:** Respondent characteristics.

**Characteristics**	**Respondent (*n* = 1,045)**
	***N*** **(%)**
Gender	
Male	507 (48.52)
Female	538 (51.48)
Age, years	
<20	133 (12.73)
20–39	386 (36.94)
40–59	388 (37.12)
≥60	138 (13.21)
Highest level of education	
Primary school and below	126 (12.06)
Junior high school	241 (23.06)
Senior high school	314 (30.05)
College degree and above	364 (34.84)
Classification of population	
First classification^†^	120 (11.48)
Second classification^‡^	203 (19.43)
Third classification^§^	363 (34.74)
Fourth classification	359 (34.35)
Location	
City	674 (64.50)
Country	371 (35.50)
Job	
Student	139 (13.30)
Office clerk	118 (11.29)
Famer	109 (10.43)
Individual operation	241 (23.06)
Medical staff	140 (13.40)
Civil servant	61 (5.84)
Teacher	87 (8.33)
Retirement	56 (5.36)
Other	94 (8.99)
Income (¥)	
<2,000	187 (17.89)
2,000–4,000	233 (22.30)
4,000–6,000	405 (38.76)
6,000–8,000	136 (13.01)
8,000–10,000	43 (4.11)
≥10,000	41 (3.92)

† *The first classification includes patients with infected COVID-19 and medical staff and managers at the front line of epidemic prevention*.

‡ *The second classification includes people who are quarantined at home or people with fever who visit hospitals*.

§ *The third classification includes people related to the first and second classifications, such as their family members, colleagues and friends, and those involved in the rear rescue response, such as onsite commanders, organization and management personnel, and volunteers*.

***¥**The fourth classification includes all populations affected by the COVID-19 except the first, second and third classification*.

### Discrete Choice Experiment Results

In Table 3, the mixed logit estimates for the total sample are reported. We found that all attributes have a significant influence on preferences for psychological interventions during the COVID-19 pandemic. The results show that the public demonstrated the strongest positive preferences for social network platforms, one-to-one form, twice-per-week visits (followed by alternating with no fixed time), family and friends as providers (followed by alternate medical staff and psychologists), and the duration for 0.5–1 h (followed by ≥1 h; all *p* < 0.01). The statistical significance of the SD coefficients for all but two of the attribute levels (phone and duration ≥1 h) confirm the existence of preference heterogeneity for most attributes.

**Table 3:** Mixed logit estimates for total sample (*n* = 1,045).

**Attributes (reference level)**	**Level**	**Coefficient (S.E)**	**SD (S.E)**
Method (face to face)	Phone	0.0530 (0.0649)	0.183 (0.201)
	Social network platform	0.882^**^ (0.0732)	1.098^**^ (0.0991)
Form (one to many)	One to one	0.209^**^ (0.0544)	0.612^**^ (0.0708)
Frequency (once every 2 weeks)	Once per week	0.0703 (0.0714)	0.583^**^ (0.106)
	Twice per week	0.952^**^ (0.0896)	1.498^**^ (0.108)
	No fixed time	0.408^**^ (0.0771)	0.719^**^ (0.130)
Provider (volunteer)	Family and friends	1.139^**^ (0.0710)	0.856^**^ (0.0987)
	Medical staff	0.551^**^ (0.0631)	0.772^**^ (0.0899)
	Psychologist	0.389^**^ (0.0664)	0.361^*^ (0.148)
Duration, hours (<0.5)	0.5–1	0.802^**^ (0.0745)	0.851^**^ (0.0904)
	≥1	0.470^**^ (0.0649)	0.158 (0.224)
Sample	1,045		
Log likelihood	−6440.3195		
Number of observations	25,080		

* *p < 0.05*,

** *p < 0.01*.

Since it is assumed that the coefficients of all attribute levels are normally distributed, the mixed logit estimates relating to the mean coefficient and SD for each attribute level were applied to calculate the distribution of preference heterogeneity. For example, the coefficient (SD) of the “family and friends” level is 1.139 (0.856), indicating that 91% of respondents exhibited a preference for psychological interventions provided by family and friends. Similarly, the results showed that 80% of respondents would prefer to be provided with psychological interventions through social network platforms.

The results of the subgroup analysis showed that the population with a primary school degree had a statistically significant preference for psychologists as providers, which is different from the population with a high school degree and college degree or above. Furthermore, the most important attribute level of the population with a primary school degree is that the duration of each intervention is 0.5–1 h (coefficient 1.064), while for the population with a middle school degree and college degree or above, the most important attribute level is that the frequency is twice per week (coefficients 1.530 and 1.409, respectively). When comparing the preferences of different population classifications, the most important attribute level of each population classification is that the frequency is twice per week. Different from other population classifications, the first classification showed a strong preference for psychologists as providers. Moreover, for the duration of each intervention ≥1 h, the preference of the first and second classification populations was not statistically significant, while that of the third and fourth classification populations was significant (refer to Table 4 for details).

**Table 4:** The results of subgroup analysis.

**(A) Group by educational level**									
**Attributes (reference level)**			**Level**	**Primary school and below**		**Junior or Senior high school**			**College degree and above**	
				**Coefficient (SE)**	**SD (SE)**	**Coefficient (S.E)**	**SD (SE)**	**Coefficient (SE)**	**SD (SE)**
Method (face to face)			Phone	0.689^**^ (0.12)	0.00168 (0.201)	0.653^**^ (0.0635)	0.395^**^ (0.122)	0.900^**^ (0.0832)	0.474^**^ (0.146)
			Multimedia	0.914^**^ (0.251)	0.00096 (0.358)	0.793^**^ (0.121)	0.181 (0.217)	0.899^**^ (0.154)	0.516^**^ (0.128)
Form (one to many)			One to one	0.248^*^ (0.0978)	0.0662 (0.546)	0.299^**^ (0.0518)	0.386^**^ (0.101)	0.372^**^ (0.0691)	0.527^**^ (0.11)
Frequency (once every 2 weeks)			Twice per week	0.800^**^ (0.249)	0.00387 (0.204)	1.530^**^ (0.122)	0.181 (0.267)	1.409^**^ (0.153)	0.0729 (0.201)
			once per week	0.908^**^ (0.142)	0.0154 (0.235)	0.562^**^ (0.0702)	0.320^*^ (0.149)	0.576^**^ (0.0898)	0.310 (0.179)
			No fixed time	−0.275 (0.389)	1.294^**^ (0.189)	1.294^**^ (0.189)	0.263 (0.236)	1.033^**^ (0.24)	0.0675 (0.546)
Provider (volunteer)			Psychologist	0.493^*^ (0.238)	0.00368 (0.231)	0.0519 (0.116)	0.0193 (0.195)	0.0741 (0.152)	0.474^**^ (0.168)
			Medical staff	0.0454 (0.262)	0.0668 (0.439)	1.010^**^ (0.13)	0.00653 (0.223)	1.245^**^ (0.164)	0.0372 (0.264)
			Friends and family	0.873^**^ (0.307)	0.686^**^ (0.22)	1.237^**^ (0.145)	0.181 (0.271)	1.094^**^ (0.182)	0.497^**^ (0.161)
Duration, hours(<0.5)			0.5-1	1.064^**^ (0.258)	0.0198 (0.181)	0.608^**^ (0.124)	0.385^**^ (0.119)	0.559^**^ (0.156)	0.245 (0.204)
			≥1	0.559^**^ (0.176)	0.0328 (0.302)	0.263^**^ (0.0834)	0.266 (0.169)	0.159 (0.107)	0.172 (0.267)
Sample			N/A	126		555		364	
Log likelihood			N/A	−862.41486		−3708.3811		−2402.8721	
Number of observations			N/A	3,024		13,320		8,736	
**(B) Group by classification of population**									
**Attributes (reference level)**	**Level**	**First classification**		**Second classification**		**Third classification**		**Fourth classification**	
		**Coefficient (SE)**	**SD (SE)**	**Coefficient (SE)**	**SD (SE)**	**Coefficient (SE)**	**SD (SE)**	**Coefficient (SE)**	**SD (SE)**
Method (face to face)	Phone	0.906^**^ (0.14)	0.382 (0.284)	0.784^**^ (0.105)	0.347 (0.213)	0.836^**^ (0.0757)	0.000668 (0.295)	0.614^**^ (0.0756)	0.218 (0.223)
	Multimedia	1.103^**^ (0.264)	0.00296 (0.177)	0.683^**^ (0.195)	0.0181 (0.288)	1.144^**^ (0.147)	0.249 (0.203)	0.722^**^ (0.15)	0.232 (0.21)
Form (one to many)	One to one	0.362^**^ (0.106)	0.157 (0.551)	0.372^**^ (0.0875)	0.420^**^(0.158)	0.326^**^ (0.0621)	0.279^*^ (0.159)	0.234^**^ (0.0651)	0.457^**^ (0.116)
Frequency (once every two weeks)	Twice a week	1.191^**^ (0.262)	0.0644 (0.385)	1.811^**^ (0.206)	0.520^**^ (0.201)	1.472^**^ (0.147)	0.00019 (0.288)	1.147^**^ (0.149)	0.0898 (0.271)
	once a week	0.951^**^ (0.157)	0.368 (0.32)	0.449^**^ (0.118)	0.31 (0.251)	0.618^**^ (0.0858)	0.115 (0.421)	0.749^**^ (0.0908)	0.494^**^ (0.135)
	No fixed time	−0.318 (0.396)	0.0331 (0.333)	1.388^**^ (0.309)	0.0183(0.311)	1.196^**^ (0.224)	0.00174 (0.225)	0.846^**^ (0.233)	0.119 (0.406)
Provider (volunteer)	Psychologist	1.003^**^ (0.254)	0.012 (0.282)	0.153 (0.194)	0.000363 (0.181)	0.227 (0.141)	0.0746 (0.303)	0.113 (0.146)	0.411^*^ (0.181)
	Medical staff	0.226 (0.284)	0.536^*^ (0.264)	1.325^**^ (0.213)	0.0423 (0.307)	1.085^**^ (0.161)	0.456^**^ (0.158)	0.524^**^ (0.162)	0.288 (0.21)
	family and Friends	1.115^**^ (0.318)	0.393 (0.321)	1.539^**^ (0.238)	0.351 (0.261)	0.916^**^ (0.176)	0.277 (0.231)	0.990^**^ (0.182)	0.512^**^ (0.156)
Time, hours (<0.5)	0.5-1	0.781^**^ (0.278)	0.137 (0.519)	0.485^*^ (0.201)	0.293 (0.203)	0.831^**^ (0.15)	0.0323 (0.336)	0.755^**^ (0.156)	0.136 (0.194)
	≥1	0.363 (0.187)	0.317 (0.334)	0.0917 (0.135)	0.0168 (0.18)	0.220^*^ (0.103)	0.311 (0.181)	0.359^**^ (0.107)	0.381^*^ (0.157)
Sample	N/A	120		203		363		359	
Log likelihood	N/A	−781.91903		−1330.121		−2350.5265		−2504.1709	
Number of observations	N/A	2,880		4,872		8,712		8,616	

* *p < 0.05,*

** *p < 0.01*.

*SD, standard deviation; SE, standard error*.

### Predicting Choice Probabilities for Different Psychological Intervention Scenarios

Supplementary 1 presents the 5 most valued psychological intervention scenarios to illustrate respondents' preferences for the factors in combination. The most ideal scenario is a one-on-one psychological intervention provided by family and friends through social network platforms, for which the frequency is twice per week and for which the duration of each intervention is 0.5–1 h. In addition, the public would prefer to increase the duration of each intervention from 0.5–1 to ≥1 h rather than change the method, frequency, and provider. However, with the same duration of each intervention (0.5–1 h), the rankings also showed that the public would accept alternating methods, frequencies, and providers.

## Discussion

To the best of our knowledge, this is the first study to explore the public's preferences for psychological interventions during the COVID-19 pandemic. In our study, the characteristics of psychological intervention programs were described by the method, form, frequency, provider, and duration. Our results demonstrated that family and friends were the most preferred providers. Furthermore, the public's preference for providers and duration was influenced by its level of education and classifications. The most ideal scenario is a one-on-one psychological intervention provided by family and friends through social network platforms, the frequency of which is twice per week, and the duration of each intervention is 0.5–1 h. Apart from the program outlined above, the public would also accept alternating social network platforms with phone calls, alternating frequencies such as twice per week with no fixed time, or alternating providers like family and friends with medical staff if the duration was not changed (0.5–1 h).

In China, the providers of psychological interventions are mostly mental health professionals (33). For example, psychological intervention teams, such as psychological intervention supervisors, psychological consultants, and psychiatrists, were established to prevent, deal with, and evaluate the potential and real mental crisis of injured people from the Lushan earthquake (34). However, in our study, most of the public (91%) during the COVID-19 pandemic has had increased preferences for family and friends as providers. This finding seems to verify the conclusion of a South Korean study, which showed that the response of patients with COVID-19 to their families is different from that of other populations (35). The reasons behind this finding may be as follows: on the one hand, COVID-19 is usually spread from person to person *via* respiratory droplets, which are expelled by speaking, sneezing, or coughing. The high risk caused by contact with strangers changes people's reactions to strangers. People are familiar with their family and friends and know with whom they have been in contact, which to some extent reduces the risk of infection. On the other hand, people who are anxious or depressed are often reluctant to seek psychological intervention due to the associated stigma (36, 37). In the study of Mythili et al. (38), one-third of respondents sought guidance for relatives and friends' psychological problems, which seems to indicate that it is feasible to provide psychological guidance to people's relatives and friends and make them a provider of psychological intervention.

At the same time, subgroup analyses revealed that the population with a low education level and the first classification population (mainly including patients with COVID-19 and medical staff and managers at the frontline of pandemic prevention) showed a strong preference for psychologists. We were unable to analyze the role of psychologists in a population with a low education level based on the current data. The study suggested that patients infected with COVID-19 and without psychiatric disorders may develop several psychiatric symptoms, including anxiety, fear, depression, and insomnia, after treatment with antiviral drugs (39). This finding may explain why psychologists are preferred by patients.

Medical staff and managers at the frontline of pandemic prevention interact directly with potentially positive or positive patients with COVID-19. They are not only working extremely hard, but they are also struggling to treat a new viral disease that is not well-understood. This situation creates a unique psychiatric burden. For instance, this study demonstrated that general distress was present in 72% of frontline healthcare workers, followed by symptoms of insomnia (34%), anxiety (45%), and depression (50%) (6). The management and scheduling of people, property, and materials are one of the main tasks for managers, such as government personnel and health administration departments, to respond to health emergencies. However, the WHO pointed out that due to the prevalence of COVID-19, the world is facing a chronic shortage of personal protective equipment, such as ventilators and masks, which brings about challenges to the work of frontline managers and may bring about an enormous psychiatric burden for managers. This psychiatric burden may lead to medical staff and managers' preferences for psychologists.

In terms of intervention methods, people are more willing to accept interventions through social network platforms or by phone than face-to-face interventions. Traditional face-to-face psychological intervention increases the risk of COVID-19 infection. Psychological interventions by telephone or through social network platforms can improve social security. One study confirmed that telehealth services are as effective as are face-to-face health services (40). In addition, Ning Wei et al. have achieved good results through internet-based integrated intervention for psychological intervention in patients with COVID-19 (41). The experience reported by Zhang et al. provides the basis for remote intervention, in which the providers of psychological interventions responded to the psychological crisis during the COVID-19 pandemic through WeChat, Huayitong, and psychological hotlines (1). The research of Mythili et al. shows that it is feasible to use a telephone to carry out psychological intervention among the public (38). Thus, social network platforms or phones should be feasible and effective in providing psychological interventions for the public during the COVID-19 pandemic with the development of 4G and 5G networks and the popularization of smartphones. The specific strategies and implementation of interventions through social network platforms or by phone should be further studied and evaluated.

The expansion of the built-up area of social network platforms lacks uniformity. For example, there are more urban internet users than rural internet users, and the number of urban users who use mobile phones to access the internet is 44% more than rural users (42), whereas almost every home in China has a telephone. Our study results showed that when ensuring the duration of each intervention (0.5–1 h), social network platforms can be alternated with phone calls, twice per week can be alternated with no fixed time, or family and friends can be alternated with medical staff. Thus, for the region or population that did not meet the most ideal scenario, our study provided choice probabilities that are predicted to be accepted by the public.

Based on the findings of our research and currently available literature, the following recommendations are made for providing psychological interventions during the COVID-19 pandemic:

1. Psychological intervention providers should include family and friends, medical staff, and psychologists. Psychological knowledge training should be carried out for people with high cognitive levels so that they can publicize psychological knowledge, guide family members and friends, and prevent the occurrence of psychological problems among the public. Psychological knowledge training for medical staff should be strengthened, and self-psychological training should be improved for people with fever or suspected infection. The ability to regulate and initiate psychological interventions should be considered, and psychologists should provide psychological guidance or interventions to people with cognitive impairment and those infected with COVID-19.

2. Remote intervention is the first choice, and network platform intervention should be effectively combined with telephone intervention.

3. One-on-one psychological interventions should be provided, the frequency of which is twice per week and the duration of which is 0.5–1 h. One-to-one intervention should be the main method, twice per week, for 0.5–1 h each time.

The findings of this study provide a reference for the formulation and revision of psychological intervention policies during the COVID-19 pandemic and the establishment of psychological intervention procedures for public health emergencies. The strengths of our study are that the sample not only is large in number (*n* = 1,045) but also was recruited from across China, which improves the objectivity of the results. This study also has certain limitations. First, like other DCEs, this study did not include all attributes. The attributes that were not included may also be very important and may affect the results to a certain extent. Second, our sampling method is convenience sampling rather than random sampling, which means that our results cannot be generalized to the whole population. Fortunately, our sample is not only large in number (*n* = 1,045) but also recruited from across the country, which alleviates this limitation to some extent. Third, in the subgroup analysis, there were certain differences in the number of people in each group, which may be due to a certain sampling bias, which in turn limits our interpretation of the results. Finally, because there are currently no studies on the preferences of the general public for psychological interventions, we cannot better compare the differences between what was available before the COVID-19 pandemic and what is currently available during the COVID-19 pandemic.

## Conclusions

The public's preferences for psychological interventions during the COVID-19 pandemic are affected by the method, form, frequency, provider, and duration. People with different levels of education or different classifications of the population have different preferences. Some suggestions for psychological interventions were put forward to provide references for the formulation of psychological intervention policies and the establishment of psychological intervention procedures in response to public health emergencies.

## Data Availability Statement

The original contributions presented in the study are included in the article/Supplementary Material, further inquiries can be directed to the corresponding authors.

## Ethics Statement

The studies involving human participants were reviewed and approved by Ethics Committee of the School of Nursing of Jilin University. Written informed consent to participate in this study was provided by the participants' legal guardian/next of kin.

## Author Contributions

H-QL: conceptualization, methodology, writing—reviewing, editing, and writing the original draft. S-XL: writing—reviewing and editing and proofreading. HX: data collection and data analyses, writing—reviewing, and editing. HY: data collection and data analyses, and writing—reviewing. X-YZ: conceptualization, methodology, writing—reviewing, and supervision. All authors contributed to the article and approved the submitted version.

## Conflict of Interest

The authors declare that the research was conducted in the absence of any commercial or financial relationships that could be construed as a potential conflict of interest.

## Publisher's Note

All claims expressed in this article are solely those of the authors and do not necessarily represent those of their affiliated organizations, or those of the publisher, the editors and the reviewers. Any product that may be evaluated in this article, or claim that may be made by its manufacturer, is not guaranteed or endorsed by the publisher.
