# Supplementary material for: The Public's Preferences for Psychological Interventions During the COVID-19 Pandemic: A Discrete Choice Experiment
Source: Front Psychiatry. 2022 Apr 27;13:805512. doi: 10.3389/fpsyt.2022.805512 (PMC9091726; doi:10.3389/fpsyt.2022.805512)
Supplement: Supplementary file 1 [file Table_1.docx]

**Supplement 1. Preference scores & predicted probabilities within the top 5 psychological intervention scenarios**

| Method | Form | Frequency | Provider | Duration, hours | Preference score | Probability | Rank |
| --- | --- | --- | --- | --- | --- | --- | --- |
| Social network platform | One on one | Twice a week | Family and friends | 0.5-1 | 3.984 | 0.104 | 1 |
| Social network platform | One on one | Twice a week | Family and friends | ≥1 | 3.652 | 0.074 | 2 |
| Social network platform | One on one | No fixed time | Family and friends | 0.5-1 | 3.438 | 0.060 | 3 |
| Social network platform | One on one | Twice a week | Medical staff | 0.5-1 | 3.396 | 0.058 | 4 |
| Phone | One on one | Twice a week | Family and friends | 0.5-1 | 3.235 | 0.049 | 5 |
